# A comparative transcriptome analysis of a wild purple potato and its red mutant provides insight into the mechanism of anthocyanin transformation

**DOI:** 10.1371/journal.pone.0191406

**Published:** 2018-01-23

**Authors:** Fang Liu, Yuanjun Yang, Jianwei Gao, Changle Ma, Yuping Bi

**Affiliations:** 1 College of Life Science, Shandong Normal University, Jinan, China; 2 Institute of Vegetables and Flowers, Shandong Academy of Agricultural Sciences/ Shandong Key Laboratory of Greenhouse Vegetable Biology / Shandong Branch of National Vegetable Improvement Center, Jinan, China; 3 Biotechnology Research Center, Shandong Academy of Agricultural Sciences, Jinan, China; Murdoch University, AUSTRALIA

## Abstract

In this study, a red mutant was obtained through *in vitro* regeneration of a wild purple potato. High-performance liquid chromatography and Mass spectrometry analysis revealed that pelargonidin-3-*O*-glucoside and petunidin-3-*O*-glucoside were main anthocyanins in the mutant and wild type tubers, respectively. In order to thoroughly understand the mechanism of anthocyanin transformation in two materials, a comparative transcriptome analysis of the mutant and wild type was carried out through high-throughput RNA sequencing, and 295 differentially expressed genes (DEGs) were obtained. Real-time qRT-PCR validation of DEGs was consistent with the transcriptome date. The DEGs mainly influenced biological and metabolic pathways, including phenylpropanoid biosynthesis and translation, and biosynthesis of flavone and flavonol. In anthocyanin biosynthetic pathway, the analysis of structural genes expressions showed that three genes, one encoding phenylalanine ammonia-lyase, one encoding 4-coumarate-CoA ligase and one encoding flavonoid 3′,5′-hydroxylasem were significantly down-regulated in the mutant; one gene encoding phenylalanine ammonia-lyase was significantly up-regulated. Moreover, the transcription factors, such as bZIP family, MYB family, LOB family, MADS family, zf-HD family and C2H2 family, were significantly regulated in anthocyanin transformation. Response proteins of hormone, such as gibberellin, abscisic acid and brassinosteroid, were also significantly regulated in anthocyanin transformation. The information contributes to discovering the candidate genes in anthocyanin transformation, which can serve as a comprehensive resource for molecular mechanism research of anthocyanin transformation in potatoes.

## Introduction

Potato (*Solanum tuberosum* L.) has been the fifth largest crop in the world. It is nutritious with a reputation of "underground apple". Red and purple-fleshed potatoes additionally contain 2 times higher anthocyanin content in comparison with white-flesh potatoes [1]. Anthocyanin, a family of water-soluble bioactive flavonoids, is in charge of producing various colors such as red, blue, and purple in plants. It can be classified into six main types: pelargonidin, cyanidin, delphinidin, peonidin, petunidin, and malvidin [2]. Potatoes with anthocyanins have benefits on health. The studies with rats demonstrate anthocyanins extracted from potatoes can significantly increase SOD activity and antioxidant capacity, and protect from injury [3, 4]. Anthocyanins in potatoes exert an anti-bacterial activity against different bacterial strains and a slight activity against three fungal strains [5].

Currently, owing to the potential advantages of anthocyanin to human health, the researches of anthocyanin biosynthesis in potato have been widely investigated. These researches are mainly concentrated on structural genes and regulation genes encoding transcription factor. In the aspect of structural genes, some structural genes have also been cloned and their functions are identified. Earlier researches show that *R* locus and *P* locus are involved in the coloration of potato tuber skin [6, 7]. *P* is epistatic to *R* [8]. *P* encodes flavonoid 3′,5′-hydroxylase (F3′5′H) [9]. *F3*′*5*′*H* is responsible for producing blue/purple petunidin-based anthocyanins and its function is verified by an immobilized transposon *dTstu1* in *F3′5′H* [10]. *R* encodes dihydroflavonol 4-reductase (DFR) [11, 12]. *DFR* causes not only an increase in red pelargonidin, but also an increase in purple petunidin in potato tubers [13, 14]. Other structural genes involved in anthocyanin biosynthesis, such as *CHS* (*CHSJ* and *CHSG*), *CHI*, *F3H* and *3GT*, are cloned from potato roots, stems, leaves, flowers and tubers [15]. *CHS* and *CHI* can cause a significant increase in pelargonidin and petunidin [16]. *3GT* can deepen tuber color and increase anthocyanin content in potato tubers [17].

In the aspect of transcription factors, there are lots of transcription factors regulating anthocyanin biosynthesis discovered in potato. The transcription factor *I* is required as the third allele locus in anthocyanin biosynthesis in the tuber skin of diploid potatoes [8, 9]. When the potato lacks the functional allele *I*, the tuber exhibits white regardless of the presence of *R* and *P*. *I* is recessively epistatic to *P* and *R* [9]. *D* is another transcription factor required for the red and purple anthocyanin in the tuber skin of tetraploid potatoes, and is allelic to *I* in diploid potatoes [7]. *D* encodes the transcription factor R2R3MYB, and co-segregates with purple and red tubers in tetraploid potatoes [18]. R2R3MYB transcription factor AN1 has an effect on promoting anthocyanin biosynthesis in tubers and leaves of potatoes. The effect can be enhanced in the presence of the co-expression factors [19]. The co-expression factors mainly include bHLH and WD40-repeat proteins. A bHLH transcription regulator is co-localized with QTL on chromosome 9 [17, 20]. With the co-expression factor StbHLH1, the effect of AN1 on promoting anthocyanin biosynthesis is significantly improved [19, 21]. WD40-repeat gene *StAN11* is cloned and significantly increases the accumulation of anthocyanin in potato tubers through its overexpression [22]. MYB, bHLH and WD40 can form the MYB-bHLH-WD anthocyanin regulatory complex to improve anthocyanin biosynthesis in potato [23].

Recently, with the development of the next-generation sequencing, transcriptome is used to comprehensively analyze the anthocyanin biosynthesis in potato. Liu carried out a comparative transcriptome analysis of white potato cultivar ‘Xin Daping’ and purple potato cultivar ‘Hei Meiren’. New versions of the pathway genes and potential transcription factors in potato anthocyanin biosynthesis were discovered [24]. Kyoungwon performed a comparative transcriptome analysis of three colored potato cultivars: light-red ‘Hongyoung’, dark-purple ‘Jayoung’ and white ‘Atlantic’. Regulatory networks of red anthocyanins and purple anthocyanin in potato were established [25].

Although some articles have been reported on the anthocyanin biosynthesis of potato, insight into the regulation of anthocyanin transformation have remained future objectives. In addition, the used materials in previous researches have different genetic backgrounds, and it possibly influence the screening of differential genes and decrease the accuracy of comparative transcriptome analysis. It is urgent to use the homologous materials to study anthocyanin transformation mechanism for enriching the metabolic pathway of anthocyanin.

In this study, we fortunately obtained a red mutant SD140 from a purple wild potato SD92. Both materials were homologous, which facilitated our comparative transcriptome analysis. In order to study the mechanism of anthocyanin transformation, we identified the main types of anthocyanins and carried out a comparative transcriptome analysis of SD140 and SD92.

## Materials and methods

### Plant sources

**Fig 1:**
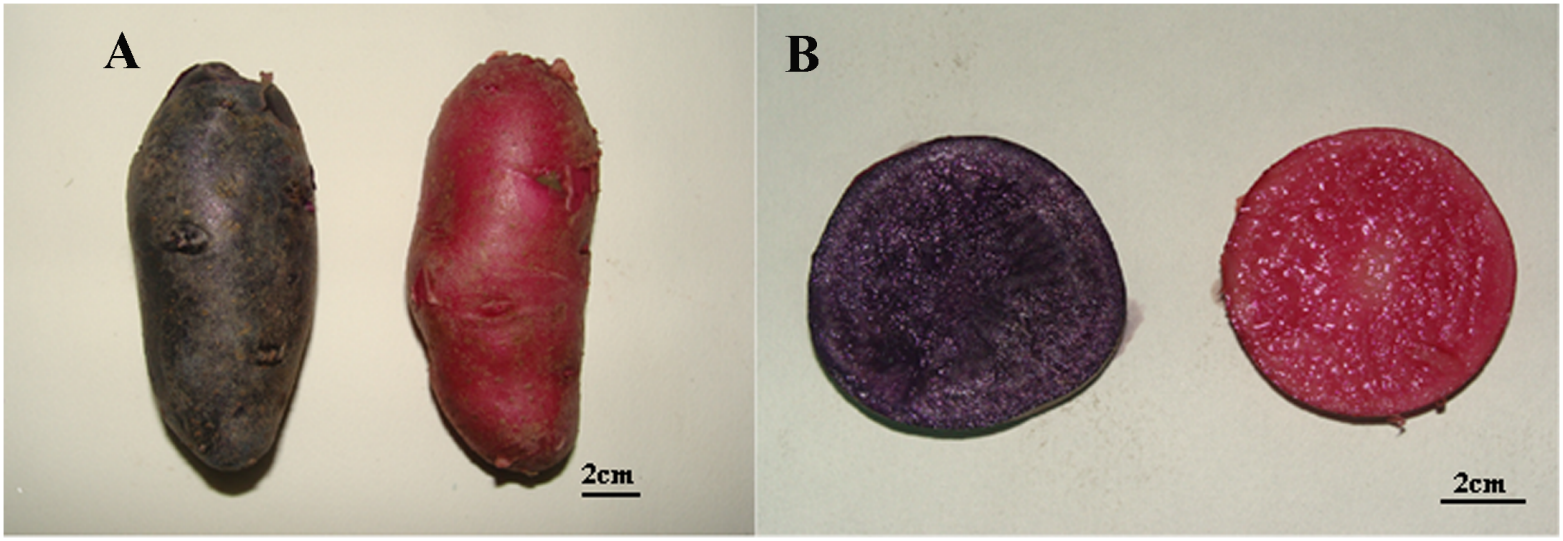
Phenotypic differences in SD92 and SD140. (A) whole tuber, (B) sectional drawing.

SD92, commonly known as Hei Jingang, is tetraploid with purple skin and purple flesh tuber. A red tuber mutant SD140 with red skin and flesh was obtained through *in vitro* regeneration of SD92 tuber slices (Fig 1). Two materials were planted in a greenhouse at 20 ± 2°C under a 16 h light/8 h dark cycle at the *Institute of Vegetables and Flowers*, *Shandong Academy of Agricultural Sciences* at the normal sowing time (August 10, 2016) in Jinan. The fresh tubers (diameter 4-5cm) were harvested, and cleaned with sterilized water. The tubers were immediately used for pigment extraction and two biological replicates were used. The tubers for mRNA extraction were frozen in liquid nitrogen and stored at -80°C, three biological replicates were used.

### Pigment extraction and purification

Potato tubers (250 g) were washed with distilled water and smashed in 500 mL of a mixture containing 50% alcohol and 1.6% citric acid. The resulting mixture was filtrated twice and centrifuged at 4000 rpm. The supernatant was transferred into a column filled with the pretreated macroporous resin AB-8 for 6 h, and the pigments absorbed in AB-8 were eluted with 95% alcohol. The eluate was concentrated with rotary evaporator at 30°C and dried in vacuo for 12 h. The laboratory protocol was deposited in protocols.io website (http://dx.doi.org/10.17504/protocols.io.ma4c2gw).

### Analytical high-performance liquid chromatography

The pigment extractions were analyzed by the ultra-performance liquid chromatography (UPLC) system (Waters, Milford, Massachusetts, USA) equipped with Waters acquity PDA detector and a BEH C18 column (2.1×100mm, 1.7μm particle size). Acetonitrile (mobile phase A) and 2% methanoic acid (mobile phase B) were used as the eluent at a flow rate of 0.3 mL/min. The gradient conditions for sample elution were as follows: 5% A 95% B, 18 min; 20% A 80% B, 20 min; 100% A, 22 min; 100% A, 22.1 min; 5% A 95% B, 25 min; 5% A 95% B. The anthocyanins were detected at 530 nm using PAD. The column temperature was kept at 45°C. The volume for each injection was 1 uL.

### Mass spectrometry

Mass spectrometry (MS) analysis was performed on a quadrupole time-of-flight (Q-TOF) mass spectrometry (WATERS MALDI SYNAPT) system. The anthocyanins were analyzed in Positive-ion (PI) mode. The optimized conditions were: the capillary and sampling cone voltage of 3.5kV and 30 V, respectively; the source block temperature of 100°C; desolvation temperature, 400°C; desolvation gas flow, 500 L/h; cone gas flow, 50 L/h; detector voltage, 1800 V. Data were gathered in the v mode, with a scan accumulation time of 1 s and the TOF data being collected between m/z 50 and 1,500. The MS-MS experiments were performed using a collision energy of 6 eV, which was optimized for each compound.

### cDNA library construction and high-throughput sequencing

According to the manufacturer’s protocol, total RNA of the sample was extracted using modified Trizol reagent (Ambion, USA). And then RNA was digested with 10U DNaseI (Thermo, USA) for 1 h at 37°C. RNA quality and purity were detected by Agilent 2100 and NanoDrop (USA). The poly (A) mRNA was enriched using Oligo (dT) magnetic beads from digested RNA and was interrupted into short fragments. Using the short mRNA fragments as templates, First-strand cDNA were synthesized using random hexamer primers. The second strand was synthesized by buffer, dNTPs, Rnase H, and DNA polymerase I. The double-stranded cDNA was obtained by PCR with illumina primers. The two-stranded cDNA was purified with magnetic beads. The cohesive ends were repaired and the base "A" was added to the 3′ end. The fragments with suitable size were selected and were enriched by PCR amplication. The cDNA library was qualified and quantified with the Agilent 2100 Bioanalyzer (USA) and ABI StepOnePlus Real-Time PCR System (USA). The qualified library was sequenced by use of Illumina HiSeqX-Ten (USA). In total, six cDNA libraries were constructed in this study. All the raw reads were acquired by RNA-seq. In order to obtain clean reads, we filtered low quality reads, adaptor sequences and the reads in which unknown base N content was too high.

We deposited all the sequence data of the six cDNA libraries into NCBI Sequence Read Archive (http://www.ncbi.nlm.nih.gov/sra/), and the accession number is SRP125987.

### Differentially expressed genes identification

All clean reads were mapped to the reference transcripts of *Solanum tuberosum* L. (PGSC_DM_v3.4_transcript-update, http://solanaceae.plantbiology.msu.edu/data) using the Bowtie2 (v2.2.5). Statistics and bioinformatics analyses were carried out on the number of clean reads mapped to reference genes and genome, sequencing saturation, and random sequencing distribution. And then gene expression levels were calculated based on the number of reads mapped to the reference sequences. To identify the differentially expressed genes (DEGs) in the two Samples, DEseq2 algorithm was used. For each gene, the average expression level in the SD92 group (Control-avg) and in the SD140 group (Treat-avg) were calculated, respectively. DEGs were screened according to the following criteria: |log2(Treatment-avg /Control-avg)| ≥ 1.00 and adjusted p-value ≤ 0.05.

### Gene Ontology (GO) enrichment and KEGG pathway enrichment analysis of DEGs

The DEGs were mapped to the corresponding GO terms and KEGG Orthology (KO) pathways. We used the R software phyper perform GO functional enrichment and KEGG functional enrichment. The p-value formula (https://en.wikipedia.org/wiki/Hypergeometric_distribution) in hypergeometric test is:

$$P=1-\overset{m-1}{\underset{i=0}{\sum }}\frac{\left(\begin{array}{c} M\\ i \end{array}\right)\left(\begin{array}{l} N-M\\ n-1 \end{array}\right)}{\left(\begin{array}{c} N\\ n \end{array}\right)}$$

Then false discovery rate (FDR) for each p-value was calculated, and the terms which FDR is not larger than 0.01 were defined as a significant enrichment.

### Transcription factor (TF) coding ability prediction in DEGs

We used getorf (EMBOSS: 6.5.7.0) to detect the ORF of each DEG, then used hmmsearch (v3.0) to align the ORF to the TF protein domain (data from PlantfDB), and finally identified TF according to the regulation based on the TF family characteristics.

### Real-time qRT-PCR validation of the genes expression difference

To validate the transcriptome sequencing results, the transcription level of DEGs in the transcriptome was verified by real-time qRT-PCR. RNA samples used for qRT-PCR were same as those used in high-throughput sequencing experiments. Gene specific primers were listed in S1 Table. 25 μL of 2× UltraSYBR Mixture (CWBIO, CHINA), 1 μL cDNA, and 50 μL of total reaction volume were applied to all the reactions following the manufacturer’s method. The qPCR reaction was performed using an iCycler iQ (Bio-Rad, Hercules, CA, USA). The qPCR conditions were 37 cycles of 15 s at 95°C and 1 min at 60°C, followed by 65°C to 95°C melting curve detection. Three biological replicates were performed and 18S RNA was used as a reference gene.

## Results

### Pigment analysis

**Fig 2:**
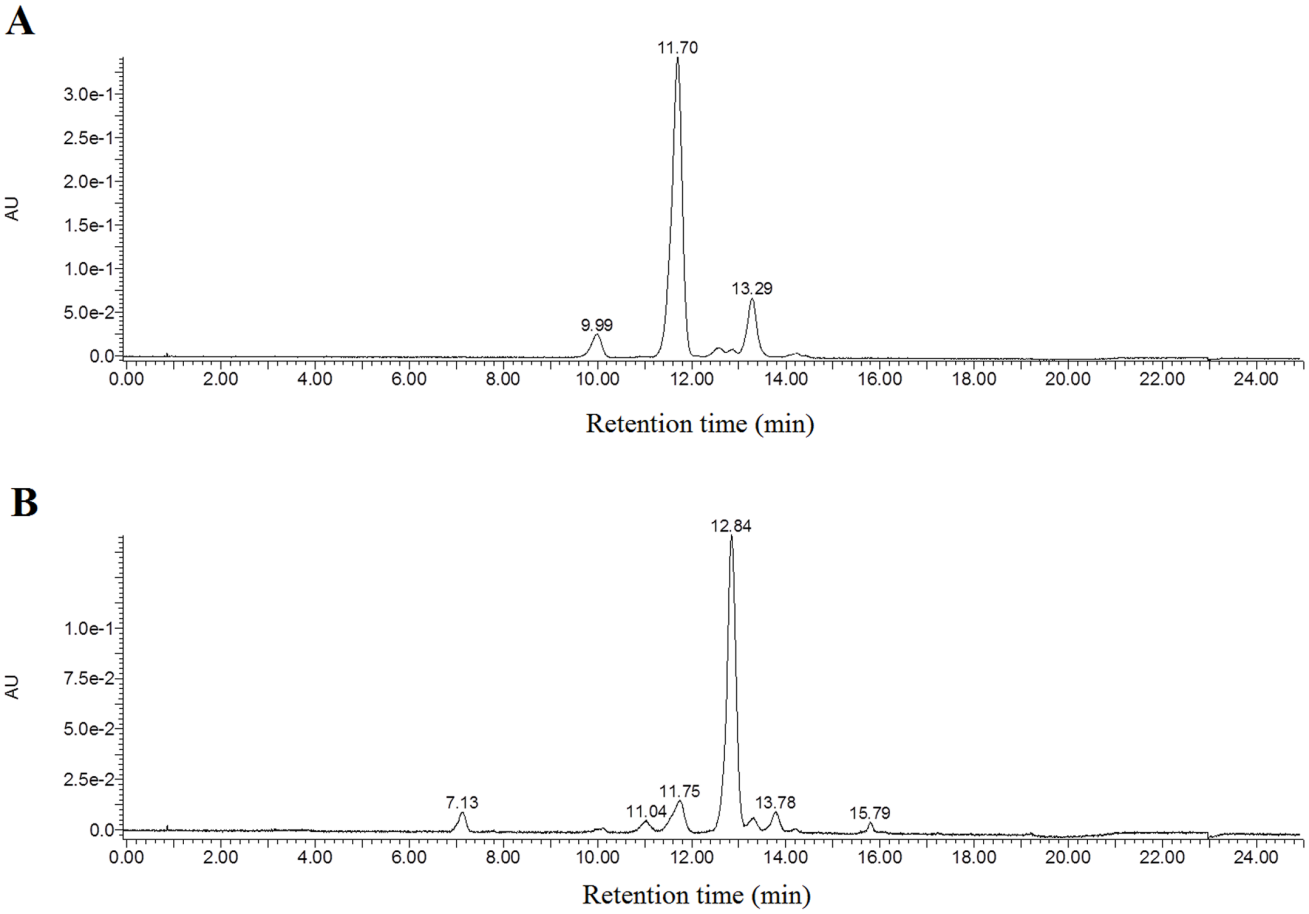
High-performance liquid chromatogram profile of anthocyanin extraction in tubers. (A) SD92, (B) SD140.

Fig 1 showed the potato tubers appearance (A) and the cross section (B) of SD140 and SD92, respectively. SD140 was obtained by the regeneration of tuber slices of SD92. After regeneration, both skin and flesh of SD92 changed from purple to red. Pure anthocyanins were obtained through pigment extraction, concentration and purification from the fresh tubers. In order to isolate the pure anthocyanins, HPLC was performed. For the anthocyanins extracted from SD140, three major independent peaks were obtained, and their retention times were located at 9.99, 11.70 and 13.29 minutes, respectively (Fig 2A). Similarly, six major peaks were acquired from SD92 extractions, and their retention times were located at 7.13, 11.04, 11.75, 12.84, 13.78, 15.79 minutes, respectively (Fig 2B). All these peaks were further identified by the quadrupole time-of-flight (Q-TOF) mass spectrometry, and the results were presented in Table 1. As for SD92, the MS analysis of the largest peak at the retention time of 11.70 minutes displayed an [M]^+^ ion at m/z 933.3 and a major fragmentation in MS^2^ at m/z 317.1. The peaks at 9.99 and 11.70 min revealed a major fragmentation in MS^2^ at m/z 317.1 (S1 Fig). The results were confirmed on the basis of HPLC retention time, elution order, spectroscopic data, MS fragmentation pattern, MS^2^, and previous findings [26–30]. The MS^2^ fragmentation of 317.1 at m/z corresponded to petunidin (Table 1). The results confirmed that the main anthocyanin in SD92 was petunidin. As for SD140, the MS analysis results of the largest peak at 12.84 min showed an [M]^+^ ion at m/z 887.3 and a major fragmentation in MS^2^ at m/z 271.1 (S2 Fig). The peaks at 7.13, 11.04, and 15.79 min exhibited a major fragmentation in MS^2^ at m/z 271.1. The MS^2^ fragmentation of 271.1 at m/z corresponded to pelargonidin. The results confirmed that the main anthocyanin in SD140 was pelargonidin (Table 1).

**Table 1:** Peak assignments of anthocyanins extracted from the tubers of SD92 and SD140.

Sample	Retention time (min)	Molecular ion [M]^+^ (*m*/*z*)	Molecular weight (Da)	MS^2^ (*m*/*z*)	Tentativel identification
**SD92**	9.99	949.3	950.3	317.1	Petunidin
		919.3	920.3	303.1	Delphinidin
	11.70	933.3	934.3	317.1	Petunidin
	13.29	917.3	918.3	301.1	Peonidin
**SD140**	7.13	579.2	580.2	271.1	Pelargonidin
	11.04	903.4	904.4	271.1	Pelargonidin
	11.75	933.4	934.4	317.1	Petunidin
	12.84	887.3	888.3	271.1	Pelargonidin
	13.78	887.4	888.4	271.1	Pelargonidin
		917.4	918.4	271.1	Pelargonidin
	15.79	725.3	725.3	271.1	Pelargonidin

### Transcriptome analysis

#### RNA sequencing and mapping of the sequence reads

Illumina sequencing technology was used to perform high-throughput RNA sequencing of SD92 and SD140, respectively. After removing the adaptor sequences and low-quality reads, 44.15–44.95 Mb clean reads were acquired from each sample. The clean reads were mapped to the genome. The mapped ratio of each sample reached 55.63%-61.49% (S2 Table). After transcripts were reconstructed, we acquired 37460 transcripts. 15232 transcripts were novel, of which 3641 were novel genes (Table 2).

**Table 2:** Summary of gene expression.

Sample	Replication	Clean reads (Mb)	Total mapping ratio (%)	Uniquely mapping ratio (%)	Total transcriptnumber	Total gene number	Novel gene number
**SD92**	1	44.42	72.92	36.24	37460	26193	3082
	2	44.15	73.58	35.88	37117	25943	3085
	3	44.46	72.90	31.30	36126	25445	3041
**SD140**	1	44.95	72.52	33.15	36835	25880	3068
	2	44.43	75.42	35.20	36747	26117	3109
	3	44.81	72.29	32.89	36530	25730	3050

To identify each gene expression in SD92 and SD140 tubers, the clean reads were mapped to the complete reference sequences, which included the reference gene sequence and novel gene. The results demonstrated that 32.39–39.51Mb clean reads were mapped in each sample. These values accounted for 72.29%-75.42% of each library (Table 2). Based on the mapped results, we counted the read coverage and the reads distribution of each transcript. The random reads distribution on the transcripts indicated that the read positions were evenly distributed in each gene (S3 Fig). Reads coverage on transcripts analysis showed that the average percentage of each gene covered by reads was approximately 50% (S4 Fig). Therefore, the sequencing data should accurately reflect gene expression and can be used for differential gene expression analysis.

#### Global gene expression pattern analysis

**Fig 3:**
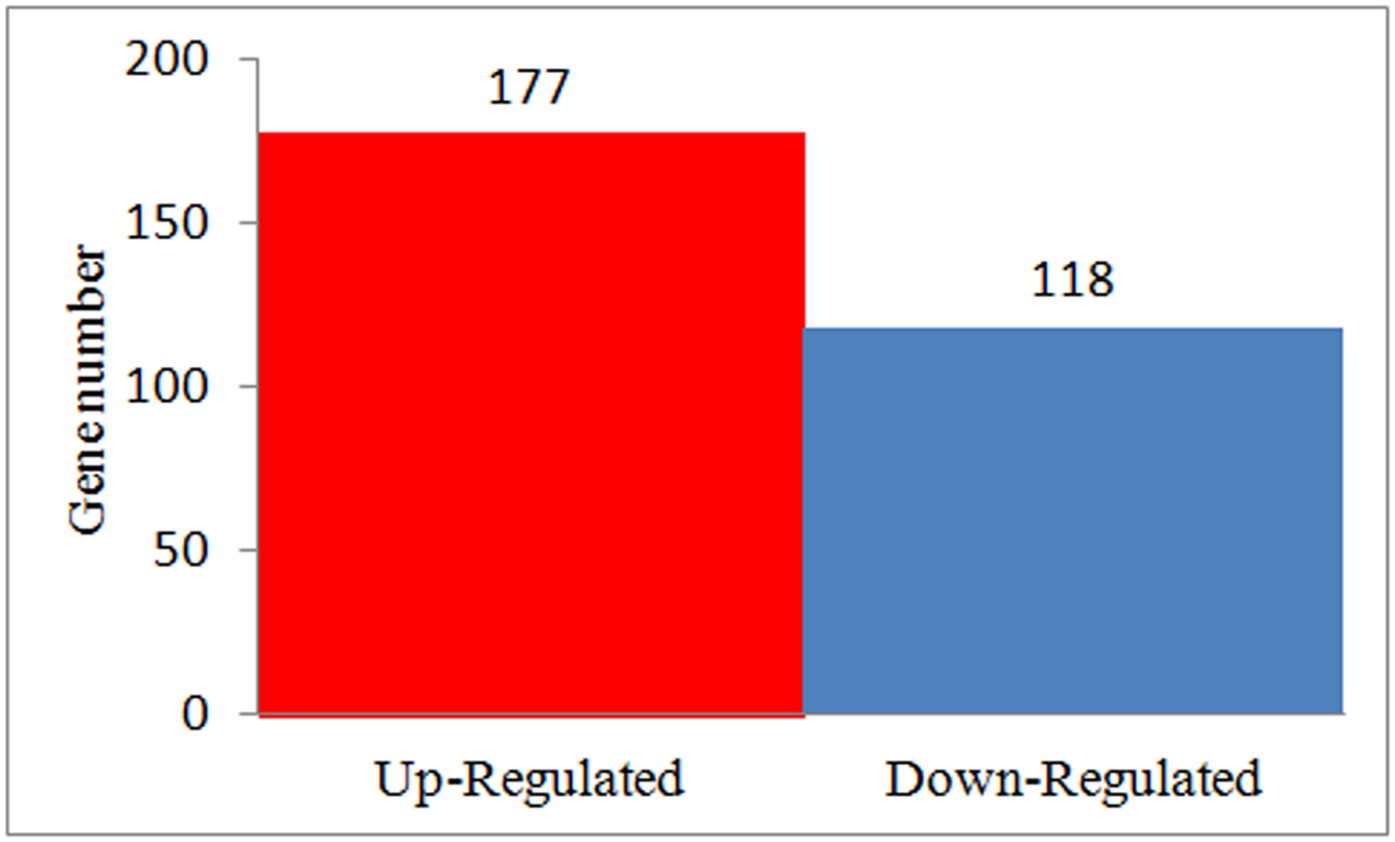
Statistics of differently expressed genes.

In SD92 and SD140, there were 22679 and 22667 genes expressed in all three biological replicates, respectively (S5 Fig). To explore the molecular mechanism involved in anthocyanin transformation, we analyzed the DEGs in three biological replicates. 267 DEGs were obtained between SD92 and SD140. Among these DEGs, 177 (60.00%) were up-regulated and 118 (40.00%) were down-regulated (Fig 3) in SD140 compared to SD92.

To facilitate the global analysis of gene expression, GO analysis of DEGs between SD92 and SD140 was performed. The results revealed 98 DEGs were classified. The DEGs were classified into the three main GO categories: biological process, cellular component and molecular function (S6 Fig). For further review of the GO classification of the DEGs, we also categorized each GO term into its sub-categories. The results indicated the sub-categories significantly enriched in the biological process category primarily contained biological regulation, localization, and response to stimulus. The DEGs in this category were associated with the regulation of catalytic activity, transcription, and macromolecule metabolic process. Cell part, cell, organelle, organelle part, membrane and membrane part were the main sub-categories belonging to the cellular component category. For the molecular function category, the main sub-categories were binding, catalytic activity, transporter activity and molecular function regulator. Catalytic activity was involved in putative RING-H2 finger protein, disulfide-isomerase protein and ras-related RABA1f protein. Proteins in the binding sub-category included beta-galactosidase, calreticulin, luminal-binding protein, kinesin-related protein, probably inactive leucine-rich repeat receptor and heat shock 70 kDa protein.

**Fig 4:**
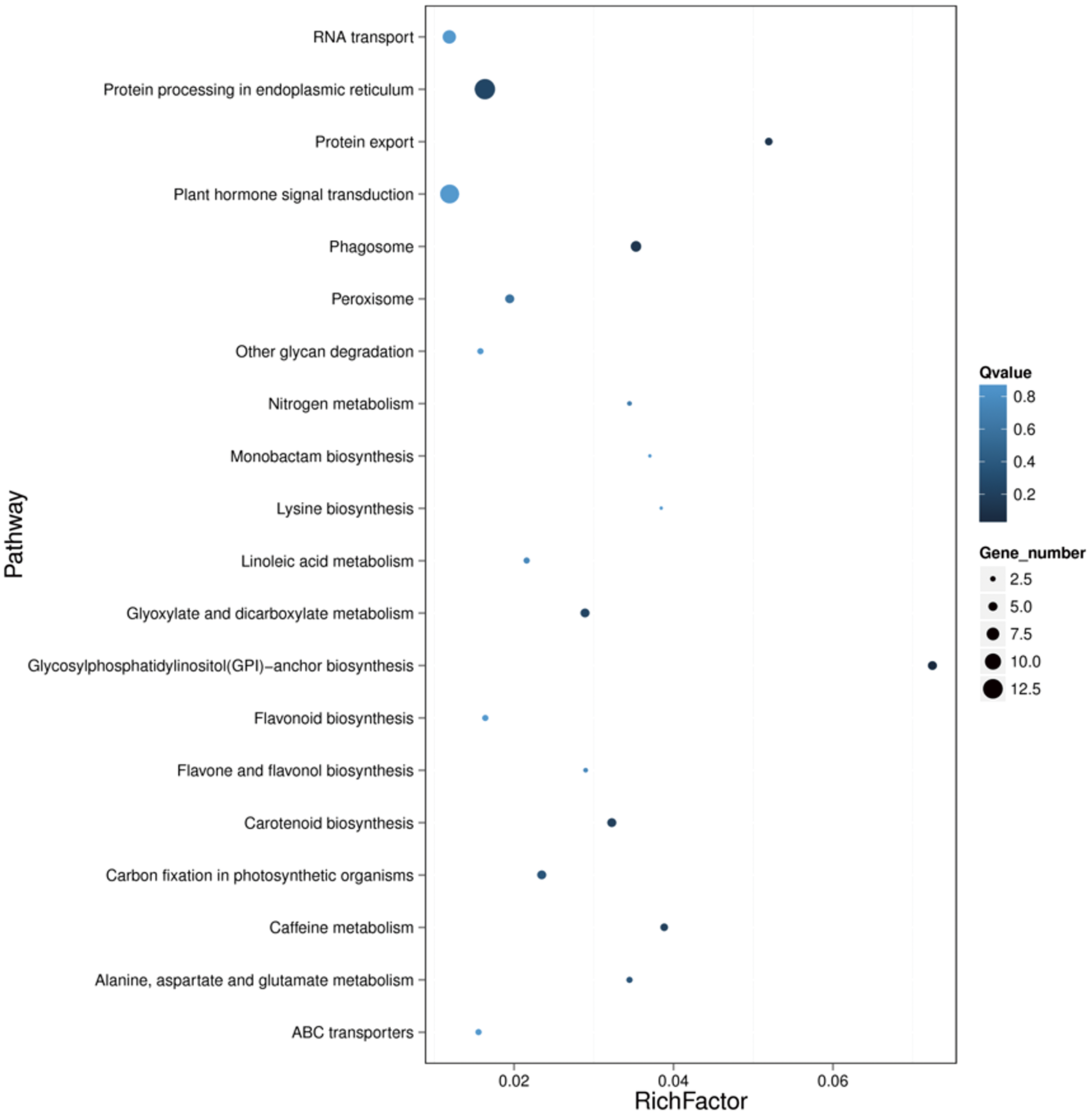
KEGG enrichment analysis of DEGs.

KEGG analysis was performed to obtain the difference in the metabolic pathway in SD92 and SD140 (Fig 4). Among the DEGs, 220 genes corresponded to KO codes, including 126 significantly up-regulated genes and 94 significantly down-regulated genes in SD140. To review the function of the DEGs, we further clustered each KO term of the DEGs. The results showed that the DEGs were mostly enriched in transport and catabolism, translation, biosynthesis of other secondary metabolites and transcription. These clusters included the following pathways: protein processing in endoplasmic reticulum (13 genes), plant hormone signal transduction (12 genes), RNA transport (8 genes), flavonoid biosynthesis (3 genes), flavone and flavonol biosynthesis (2 genes), phenylpropanoid biosynthesis (7 genes) and spliceosome (7 gens).

#### Gene expression changes of anthocyanin biosynthesis in potato

**Fig 5:**
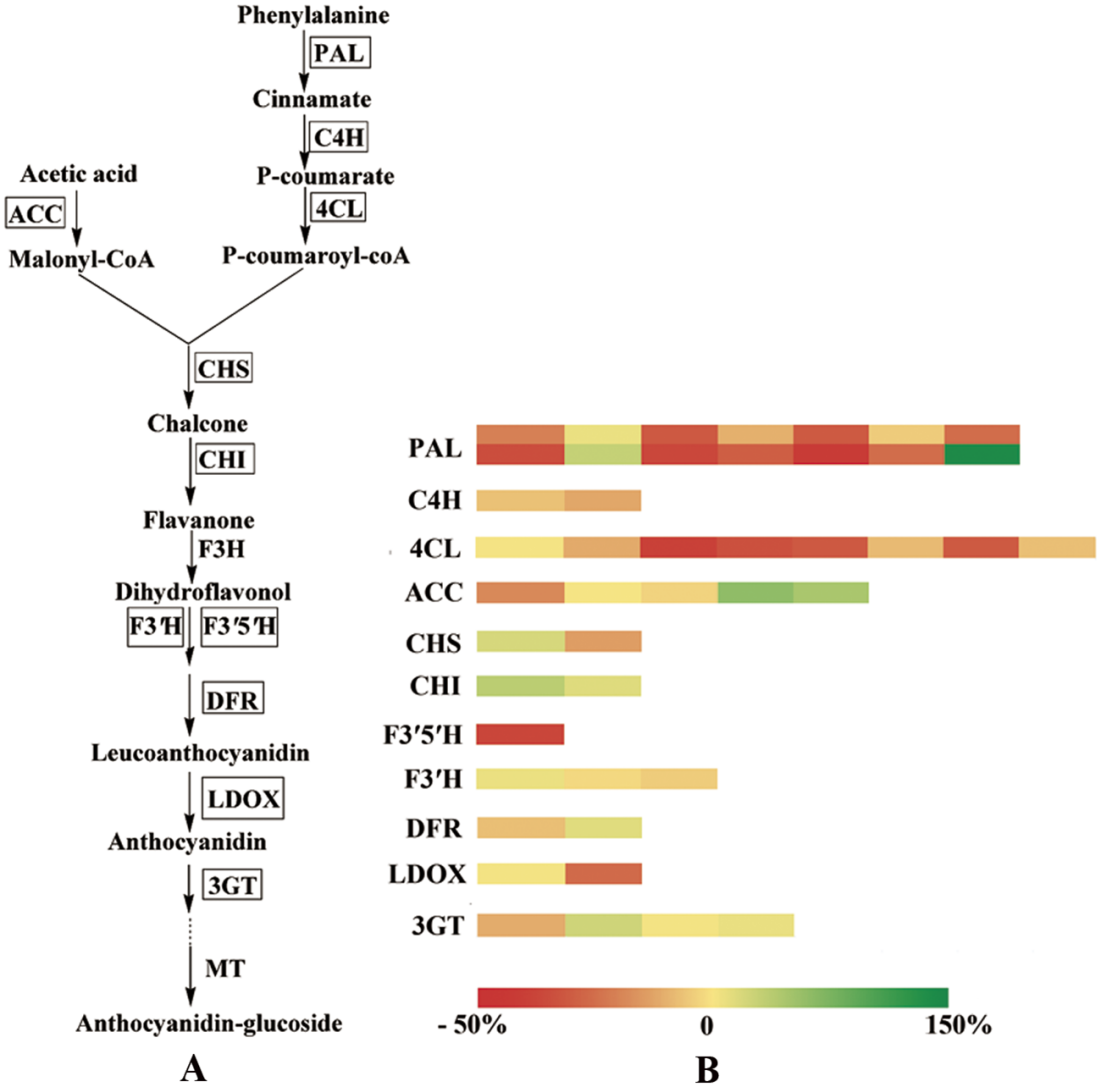
Gene expression levels of anthocyanin biosynthesis in potato tubers. (A) The metabolic pathway of anthocyanin biosynthesis; the genes encoding enzymes enclosed in boxes were detected in this transcriptome, (B) heatmap of the rate of change of gene expression.

Considering that the main type of anthocyanins in SD92 was different from SD140, and that the results of KEGG enrichment of DEGs showed differences in flavonoid metabolic pathways between SD92 and SD140, we selected 41 genes to investigate the pathway of anthocyanin biosynthesis. Of the selected genes, 8 were new genes. The criterion for selection was that the gene expression level was not less than 20 reads at least in one material (Fig 5 and S3 Table). The 41 genes mainly encoded the following enzymes: phenylalanine ammonia-lyase (PAL), 4-coumarate-CoA ligase (4CL), chalcone synthase (CHS), flavonoid 3′,5′-hydroxylase (F3′5′H), flavonoid 3′-hydroxylase (F3′H), dihydroflavonol 4-reductase (DFR), leucoanthocyanidin dioxygenase (LDOX), anthocyanidin 3-O-glucosyltransferase (3GT). These enzymes had two characteristics. One was that these enzymes possessed isozymes. Phenylalanine ammonia-lyase included two isozymes: PAL and PAL1. Chalcone synthase mainly included two isozymes: CHS1B, CHS2. 4-Coumarate-CoA ligase included 4CL2 and 4CL. The other was that these enzymes belonged to a multi-gene family. PAL and 4CL families comprised 14 and 9 genes, respectively. The differences of 41 gene expression levels in anthocyanin biosynthetic pathway were investigated according to p-value. If p-value ≤ 0.05, the gene was significantly regulated. The result showed the expression of four genes significantly changed, and other genes almost didn’t significantly changed. Four changed genes included two genes encoding PAL, one gene encoding 4CL, one gene encoding F3′5′H. To investigate the change range of these significantly changed genes, we calculated the rate of change by using the following formula: (Treatment-avg—Control-avg)/ Control-avg ×100% (S3 Table). The results indicated that in SD140 one gene encoding PAL was up-regulated by 144%, another gene encoding PAL was down-regulated by 42.58%; one gene encoding 4CL was down-regulated by 39.83%; one gene encoding F3′5′H was down-regulated by 37.66%.

#### Analysis of transcription factors in DEGs

Transcription factor is a kind of protein binding the specific nucleotide sequence of a gene and regulates the transcription of the related gene. The GO analysis indicated that there existed differences in transcription and DNA binding between SD92 and SD140. To determine the molecular mechanism of anthocyanin transformation, we examined the differential expression of transcription factors between SD92 and SD140. The results revealed the transcription factors were encoded by 18 DEGs (Table 3). These transcription factors belonged to 12 families. Alfin family, bZIP family, C3H family, FHA family, GRAS family, LOB family, MADS family were down-regulated in SD140 and were mainly involved in histone acetyltransferase, transcription factor HBP-1, zinc finger protein ZAT11, zinc finger CCCH and uncharacterized protein. S1Fa family, Tify family, zf-HD family were up-regulated in SD140 and were mainly involved in vacuolar amino acid transporter YPQ2, plastidic glucose transporter, zinc-finger homeodomain protein 9 and protein RADIALIS. The AP2-EREBP family contained AP2 ethylene-responsive transcription factor ANT and ethylene-responsive transcription factor 4-like. ANT was up-regulated and 4-like was down-regulated. The MYB family comprised myb-related protein MYBAS1 and protein RADIALIS 3. MYBAS1 was up-regulated, and RADIALIS 3 was down-regulated in SD140.

**Table 3:** Transcription factors analysis in DEGs.

TF	#Gene	Function	Up/Down- Regulation
**Alfin-like**	BGI_novel_G000417	histone acetyltransferase HAC1-like isoform X1	Down
**AP2-EREBP**	PGSC0003DMG400004921	AP2-like ethylene-responsive transcription factor ANT	Up
**AP2-EREBP**	PGSC0003DMG400022823	ethylene-responsive transcription factor 4-like	Down
**bZIP**	PGSC0003DMG400026364	transcription factor HBP-1b(c38)-like isoform X1, X3, X6, X2	Down
**C2H2**	PGSC0003DMG400030803	protein TRANSPARENT TESTA 1-like, RNA-binding protein 24-like	Up
**C2H2**	PGSC0003DMG400015534	zinc finger protein ZAT11-like	Down
**C3H**	BGI_novel_G003371	zinc finger CCCH domain-containing protein 24	Down
**FHA**	BGI_novel_G001032	uncharacterized protein LOC102601992	Down
**GRAS**	PGSC0003DMG400029921	scarecrow-like protein 8	Down
**LOB**	PGSC0003DMG400008649	LOB domain-containing protein 4	Down
**MADS**	PGSC0003DMG400022867	uncharacterized protein LOC102601973	Down
**MYB**	PGSC0003DMG400028282	myb-related protein MYBAS1	Up
**MYB**	PGSC0003DMG400015461	protein RADIALIS	Down
**S1Fa**	PGSC0003DMG400010043	probable vacuolar amino acid transporter YPQ2	Up
**S1Fa**	PGSC0003DMG400016530	probable plastidic glucose transporter 3	Up
**Tify**	PGSC0003DMG400001178	protein TIFY 5A	Up
**Tify**	BGI_novel_G002514	protein TIFY 5A	Up
**zf-HD**	PGSC0003DMG400033694	zinc-finger homeodomain protein 9	Up

#### Analysis of plant hormone signal transduction in DEGs

Hormones are growth regulators, which affect plant germination, rooting, flowering, fruiting, sex determination, dormancy and shedding. The KEGG analysis indicated that there were differences in plant hormone signal transduction between SD92 and SD140. In order to deeply analyze the molecular mechanism of anthocyanin transformation, the genes expression differences in hormone signal transduction between SD92 and SD140 were investigated. The results indicated that 12 genes expression in hormone signal transduction exhibited significant change (Table 4). The hormone signal transduction included GA, ABA, IAA, BR, Ethylene and Salicylic acid signal transduction. As for SD140, in GA signal transduction, one gene encoding scarecrow-like protein 8 was significantly down-regulated; in ABA signal transduction, one gene encoding abscisic acid receptor PYL4 was down-regulated; in BR signal transduction, one gene encoding D-type cyclin family 3 subgroup 3 and one gene encoding inactive leucine-rich repeat receptor-like protein kinase IMK2 were up-regulated, one gene encoding BRI1 kinase inhibitor 1 and one gene encoding LRR receptor-like serine/ threonine-protein kinase At2g24230 were down-regulated.

**Table 4:** Plant hormone signal transduction analysis in DEGs.

Hormone	GeneID	Kegg Orthology	Nr Description	Up/Down- Regulation
**Auxin**	PGSC0003DMG400008504	K13946	auxin transporter-like protein 2	Up
**Auxin**	PGSC0003DMG402002635	K14484	IAA15	Down
**Auxin**	PGSC0003DMG400005338	K14484	auxin-responsive protein IAA	Down
**Auxin**	BGI_novel_G000882	K14486	Auxin response factor 8	Down
**Brassinosteroid**	PGSC0003DMG400008001	K14505	D-type cyclin family 3 subgroup 3	Up
**Brassinosteroid**	PGSC0003DMG400013198	K04733,K20359,K04730,K13415	inactive leucine-rich repeat receptor-like protein kinase IMK2	Up
**Brassinosteroid**	PGSC0003DMG400008020	K14499	BRI1 kinase inhibitor 1	Down
**Brassinosteroid**	PGSC0003DMG400028478	K04730, K13415	LRR receptor-like serine/ threonine—protein kinase At2g24230	Down
**Ethylene**	PGSC0003DMG400021341	K14514	sucrose-phosphatase 1 isoform X2 lycopersicum]	Down
**Abscisic acid**	PGSC0003DMG400023949	K14496	abscisic acid receptor PYL4—like	Down
**Salicylic acid**	PGSC0003DMG400026364	K14431	transcription factor HBP-1b (c38)—like isoform X6,X2,X1, X3	Down
**Gibberellin**	PGSC0003DMG400029921	K14777, K14494	scarecrow-like protein 8	Down

#### Validation of RNA-Seq-based gene expression by qRT-PCR

**Fig 6:**
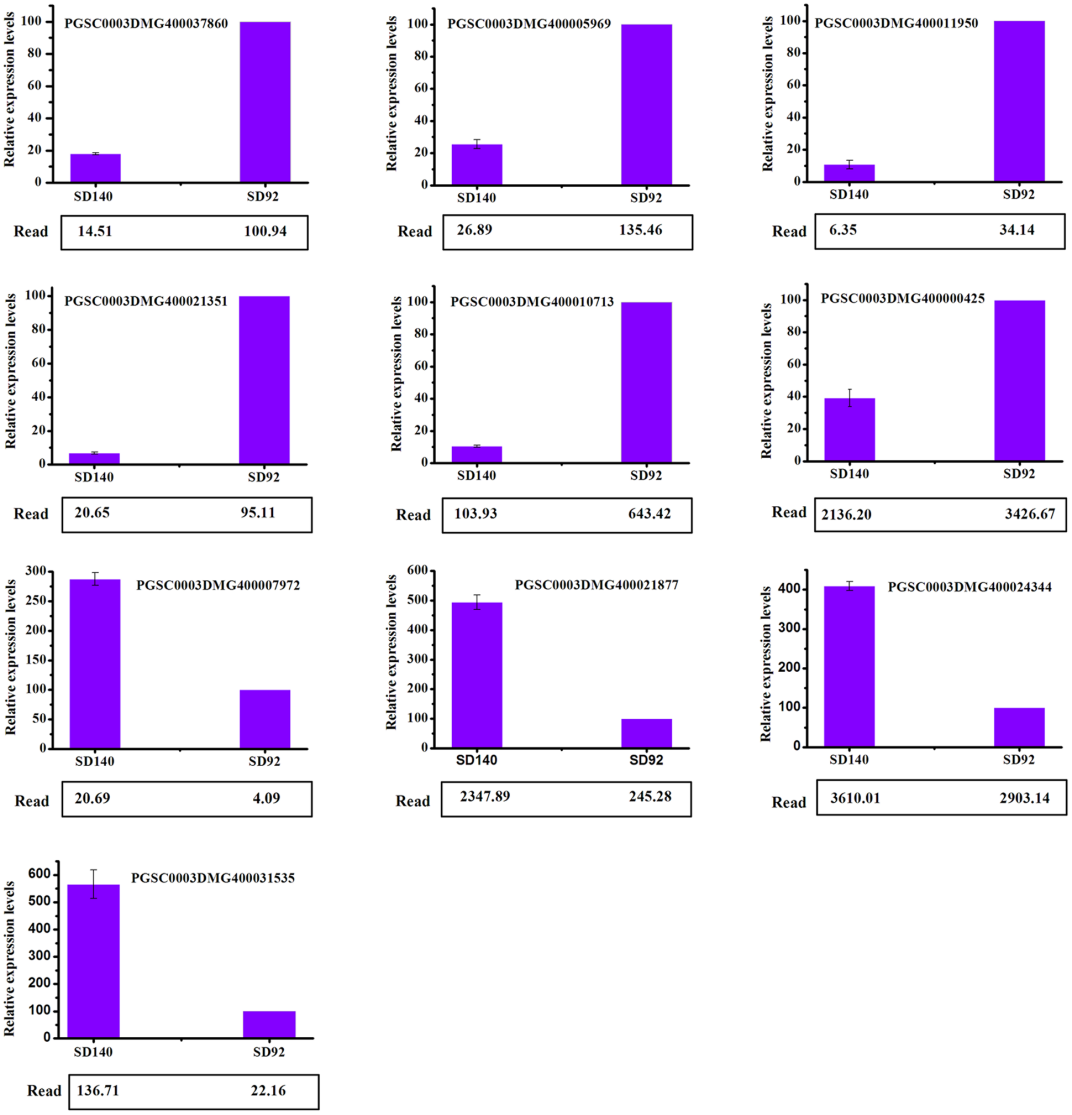
qRT-PCR verification of expression of selected genes.

To validate the RNA-Seq results, a qRT-PCR analysis was performed on 10 genes. These genes included eight randomly selected DEGs and two genes in the anthocyanin metabolic pathway. Eight randomly selected DEGs were involved in DNA-directed RNA polymerase, ubiquitin-protein ligase, ferredoxin, translation initiation factor, xyloglucosyl transferase and ABA 8′-hydroxylase. Two genes in the anthocyanin biosynthetic pathway encoded F3′5′H and LDOX. The qRT-PCR result indicated that all 10 genes expression pattern was accordant with the RNA-Seq date (Fig 6).

## Discussion

### Effect of structural genes expression differences on anthocyanin transformation in anthocyanin biosynthesis pathway

Anthocyanin biosynthesis pathway has been elucidated in many plant species. Different species have different structural genes. Many structural genes have been cloned from model species, such as maize and petunia [17]. In this study, the structural genes *PAL*, *ACC*, *4CL*, *C4H*, *CHS*, *CHI*, *F3′H*, *F3′5′H*, *ANS*, *DFR* and *3GT* were detected by analyzing the transcriptome of SD92 and SD140. The results indicated that the expressions levels of *F3′H* were both low in two materials. *F3′H* is responsible for biosynthesis of peonidin [14]. So the predicated peonidin levels were low in the two materials, which were consistent with our HPLC-MS analysis. These results further confirmed that *F3′H* was responsible for the biosynthesis of peonidin in potato.

Additionally, the differential expression analysis of structural genes in anthocyanin biosynthesis revealed that one *PAL*, one *4CL* and one *F3′5′H* were significantly down-regulated in SD140; one *PAL* was significantly up-regulated. *PAL* and *4CL* belong to anthocyanin biosynthetic upstream genes, and they are involved in multiple metabolic pathways [31, 32]. These genes expression changes may not cause changes in anthocyanin biosynthesis. We paid more attention to the gene change in downstream. *F3′5′H* belongs to anthocyanin biosynthetic downstream gene. *F3′5′H* plays a key role in regulating different types of anthocyanin biosynthesis [14]. We found that it was significantly down-regulated in anthocyanin transformation.

### Effect of *F3′5′H* on anthocyanin transformation

In this work, petunidin-3-*O*-glucoside and pelairgonidin-3-*O*-glucoside were found to be responsible for purple tuber and red tuber, respectively, and these findings were consistent with previous results [9, 15]. F3′H and F3′5′H, belonging to the cytochrome P-450 total branch, are key enzymes controlling different branches of anthocyanin biosynthesis in some species [14]. When both F3′H and F3′5′H are present, F3′5′H is predominant. In other words, F3′5′H not only promotes anthocyanins synthesis pathway to 3′ 5′-hydroxylated anthocyanins but also stops the biosynthetic pathway to 3′-hydroxylated anthocyanins [33, 34]. Besides, *F3′5′H* expression was much higher and *F3′H* expression was much lower, so *F3′5′H* had a main effect on the color of potato tuber. F3*′*5*′*H controls the biosynthesis of petunidin-based anthocyanins [17]. Silenced *F3′5′H*, inactivation and degeneration of F3*′*5*′*H can decrease petunidin-based anthocyanins [35, 36]. The decreased petunidin in SD140 was caused by the decreased expression of F3′5′H, which was verified by qRT-PCR. Moreover, the main type of anthocyanin in the purple tubers and the red tubers were petunidin and pelargonidin, respectively. Therefore, the transformation from petunidin to pelargonidin in tuber was related to the decrease of *F3′5′H* expression level and dihydrokaempferol directly synthesize pelargonidin [14]. So it was possible that the transcript of *F3′5′H* was influenced.

### Effect of transcription factors on anthocyanin transformation

Transcription factors are adaptor molecules. It can be used to detect regulatory sequences in the DNA and target the assembly of protein complexes controlling gene expression [37]. Transcription factors play important roles on controlling plant development and differentiation. In this study, the transcription factor families, including AP2-EREBP family, C2H2 family, MYB family, S1Fa family, Tify family, GRAS family, Alfin family, C3H family, FHA family, LOB family, MADS family and zf-HD family, significantly changed during anthocyanin transformation.

In plants, R2R3MYB family, a Myb-type transcription factor, is closely related to flavonoid metabolic pathways. The R2R3MYB family includes a lot of subfamilies in different species. The subfamilies, such as Stan2, VvMYBPA1, VvMYB5b and VvMYB5a, can regulate anthocyanin biosynthesis by activating structural genes. All the activated genes include *F3*′*5*′*H* [11, 38, 39]. There are also inhibitory factors in the MYB family, such as SlTRY (CPC) and MYB182. They inhibit the anthocyanin biosynthesis [40, 41]. In our study, the expressions of the genes encoding the MYB family members were significantly different in potatoes with different types of anthocyanins. It was possible that these MYB family members regulated anthocyanin transformation from petunidin to pelargonidin by regulating *F3*′*5*′*H*, which switched from 3′-hydroxylation to 3′5′-hydroxylation anthocyanin synthesis pathway. In MYB family, MYBAS1 was up-regulated, which suggested it promoted the transformation from petunidin to pelargonidin by repressing *F3*′*5*′*H* expression. RADIALIS 3 was down-regulated, which suggested it inhibited the transformation from petunidin to pelargonidin.

Besides, bZIP family also played an important role in anthocyanin transformation. bZIP, an alkaline leucine zipper transcription factor, comprised a basic part and a leucine zipper part [42]. bZIP transcription factors G/HBF-1, LONG HYPOCOTYL 5 (HY5) and HY5 HOMOLOG (HYH) can increase the structural genes expression of *CHS*, *CHI*, *F3H* and *DFR* and promote anthocyanin biosynthesis by binding the promoter of the structural genes [43, 44, 45]. bZIP transcription factors also improve anthocyanin biosynthesis by binding the R2R3MYB transcription factor PAP1 promoter region [46, 47]. R2R3MYB can regulate the expression of *F3*′*5*′*H*, which regulates transformation from petunidin to pelargonidin [36]. Therefore, bZIP can regulate anthocyanin transformation from petunidin to pelargonidin by *F3*′*5*′*H*. In this study, a gene encoding bZIP transcription factor HBP-1b (c38) -like isoform X1, X3, X6, X2 was down-regulated. It suggested that the HBP-1b (c38) inhibited the transformation from petunidin to pelargonidin by MYB.

MADS, a transcription factor, consists of the MADS-box, the I, the K and the C regions [48]. MADS family is closely related to anthocyanin biosynthesis [49–51]. MADS transcription factors increase anthocyanin biosynthesis by activating the genes encoding CHS, CHI, F3H, DFR, ANS and UFTG [49, 52]. MADS transcription factor *VmTDR4* plays an important role in accumulation of anthocyanins through the up-regulation of R2R3MYB family [53]. R2R3MYB can regulate anthocyanin transformation through *F3*′*5*′*H* [36]. So MADS can regulate anthocyanin transformation through *F3*′*5*′*H*. In this study, a gene encoding MADS was significantly down-regulated in SD140. It was possible that the MADS suppressed the transformation from petunidin to pelargonidin through regulating *F3*′*5*′*H*.

In this study, the transcription factors were significantly regulated, such as AP2-EREBP family, C2H2 family, S1Fa family, Tify family, GRAS family, Alfin family, C3H family, FHA family, LOB family, and zf-HD family. The functions of these transcription factors were worth further research.

### Effect of plant hormone on anthocyanin transformation

Plant hormone can influence anthocyanin biosynthesis. GA signal is involved in anthocyanin biosynthesis pathway. GA can decrease anthocyanin accumulation under low temperature or phosphate starvation [54, 55]. The depression effect under low temperature was determined by two bZIP transcription factors (HY5 and HYH) [54]. In this study, one DEG encoding scarecrow-like protein 8 in GA signal pathway, and one DEG encoding bZIP transcription factor HBP-1b(c38) were both down-regulated. The results suggested that scarecrow-like protein 8 inhibited the transformation through HBP-1b(c38). ABA can independently improve anthocyanin accumulation and also has a synergic effect with sugar [56, 57]. ABA regulates anthocyanin biosynthesis via MYB, bHLH and bZIP [58–60]. In this work, one DEG with the function of ABA receptor PYL4 in ABA signal pathway was down-regulated. Additonallly, the aforementioned analysis indicated that MYB and bZIP regulated anthocyanin transformation. Therefore, it could be concluded that ABA receptor PYL4 inhibited the transformation from petunidin to pelargonidin by MYB and bZIP. BR also affects JA-induced anthocyanin accumulation by the WD-repeat/MYB/bHLH transcriptional complexes [61]. In current work, D-type cyclin family 3 and inactive leucine-rich repeat receptor- like protein kinase IMK2 were up-regulated, BRI1 kinase inhibitor 1 and LRR receptor serine/threonine-protein kinase At2g24230 were down-regulated. Besides, the above-mentioned findings confirmed that MYB and bZIP regulated anthocyanin transformation. As a result, it could be infered that with the aid of the MYB and bZIP, the two up-regulated BR response proteins inhibited anthocyanin transformation and the two down-regulated BR response proteins promoted anthocyanin transformation.

Moreover, the response proteins in signal transduction of IAA and ethylene were significantly regulated in anthocyanin transformation in this work, The functions of these hormone response proteins were worth further research.

## Conclusion

**Fig 7:**
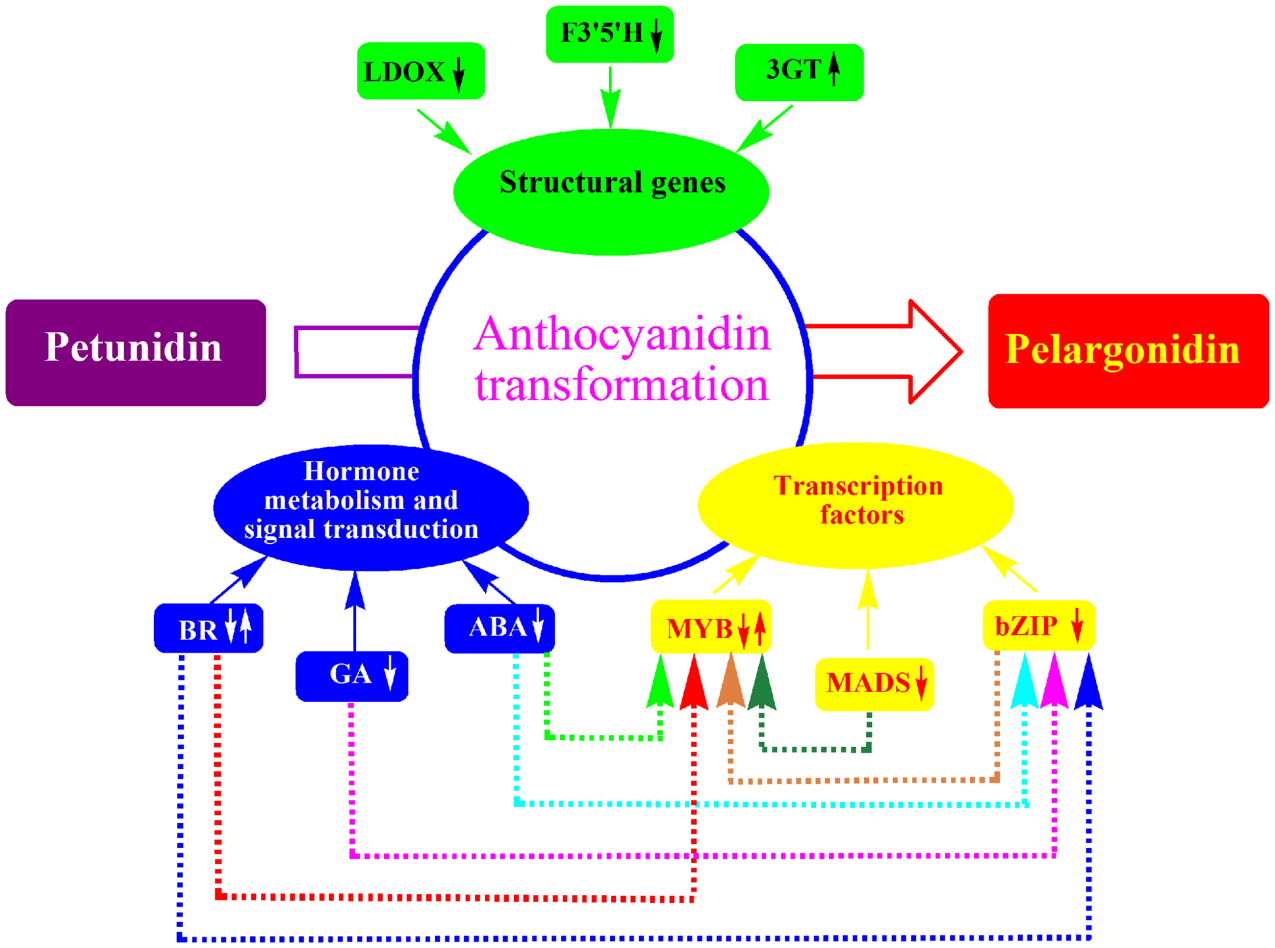
A complex that regulates the anthocyanin transformation in potato tuber.

In this study, both pigments and the comparative transcriptome analysis of purple potato tuber and its red mutant were carried out. It revealed the decreased expression level of *F3′5′H* was mainly responsible for anthocyanin transformation from petunidin to pelargonidin in potato tubers. Transcription factors bZIP family, MSD family regulated the transformation through MYB family, which regulated *F3′5′H* expression. Response proteins of ABA, GA, BR also affected the transformation through transcription factors.The structural genes, transcription factors and their related factors formed a complex that affected the transformation from petunidin to pelargonidin in potato tuber (Fig 7). The transcriptome analysis provided valuable information for anthocyanin transformation.

## Supporting information

S1 Fig Time-of-flight mass spectrum of every peak of SD92. (TIF)

S2 Fig Time-of-flight mass spectrum of every peak of SD140. (TIF)

S3 Fig Reads distribution on -transcripts. SD140:a,b,c. SD92: d,e,f.
(TIF)

S4 Fig Reads coverage on transcripts. SD140:a,b,c. SD92: d,e,f.
(TIF)

S5 Fig Venn diagram analysis between samples. A: SD92, B: SD140.
(TIF)

S6 Fig GO enrichment analysis of DEGs. A threshold of corrected p value ≤ 0.001 was used to judge the significantly enriched GO terms in DEGs.
(TIF)

S1 Table List of primers used for validation of the differently expressed genes. (DOC)

S2 Table Summary of mapping results (mapping to genome). (DOC)

S3 Table Expression of genes involved in anthocyanin biosynthesis. (DOC)
