# Supplementary figures and images for: A comparative transcriptome analysis of a wild purple potato and its red mutant provides insight into the mechanism of anthocyanin transformation
Source: PLoS One. 2018 Jan 23;13(1):e0191406. doi: 10.1371/journal.pone.0191406 (PMC5779664; doi:10.1371/journal.pone.0191406)

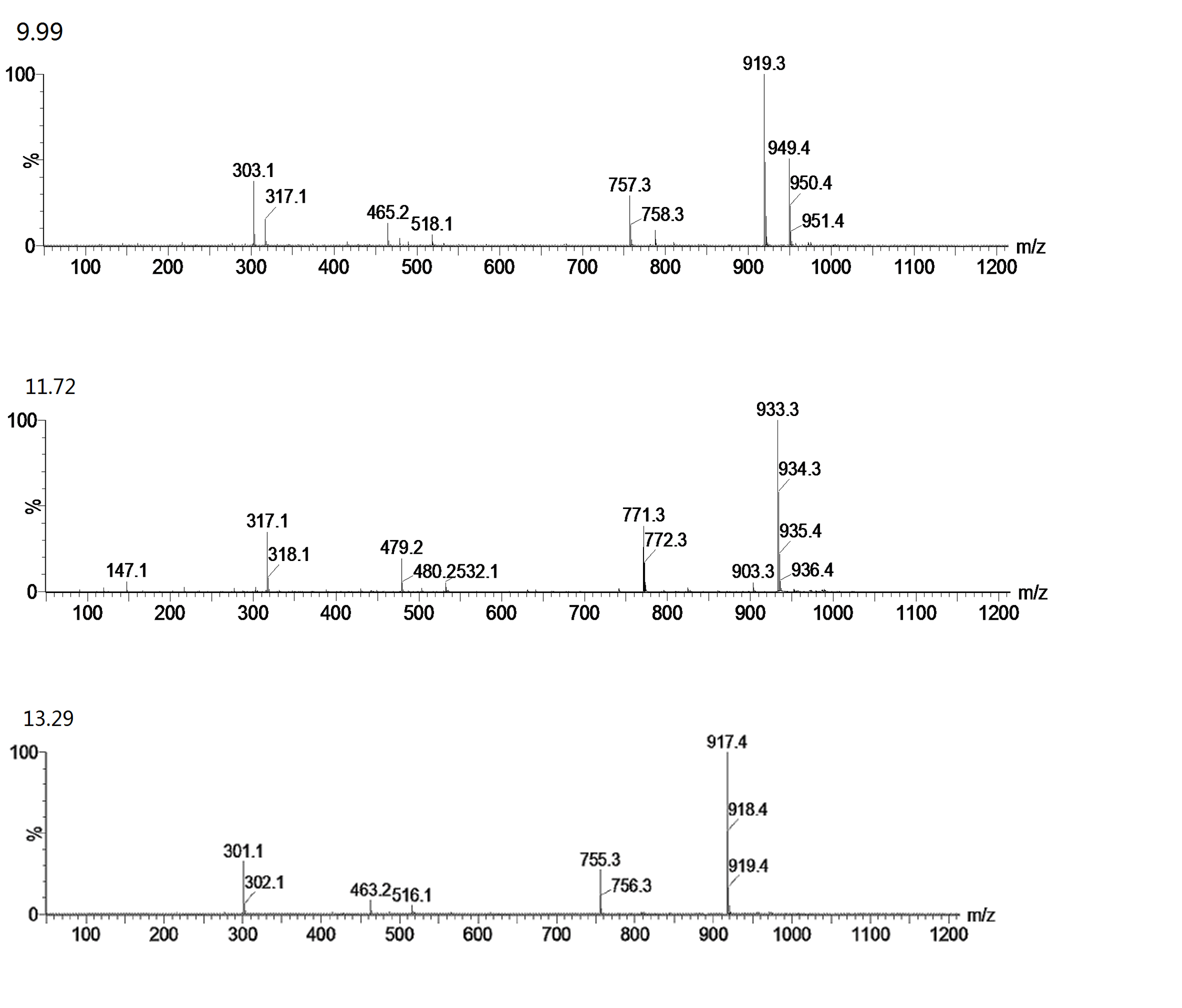

Supplement: S1 Fig — (TIF) [file pone.0191406.s001.tif]

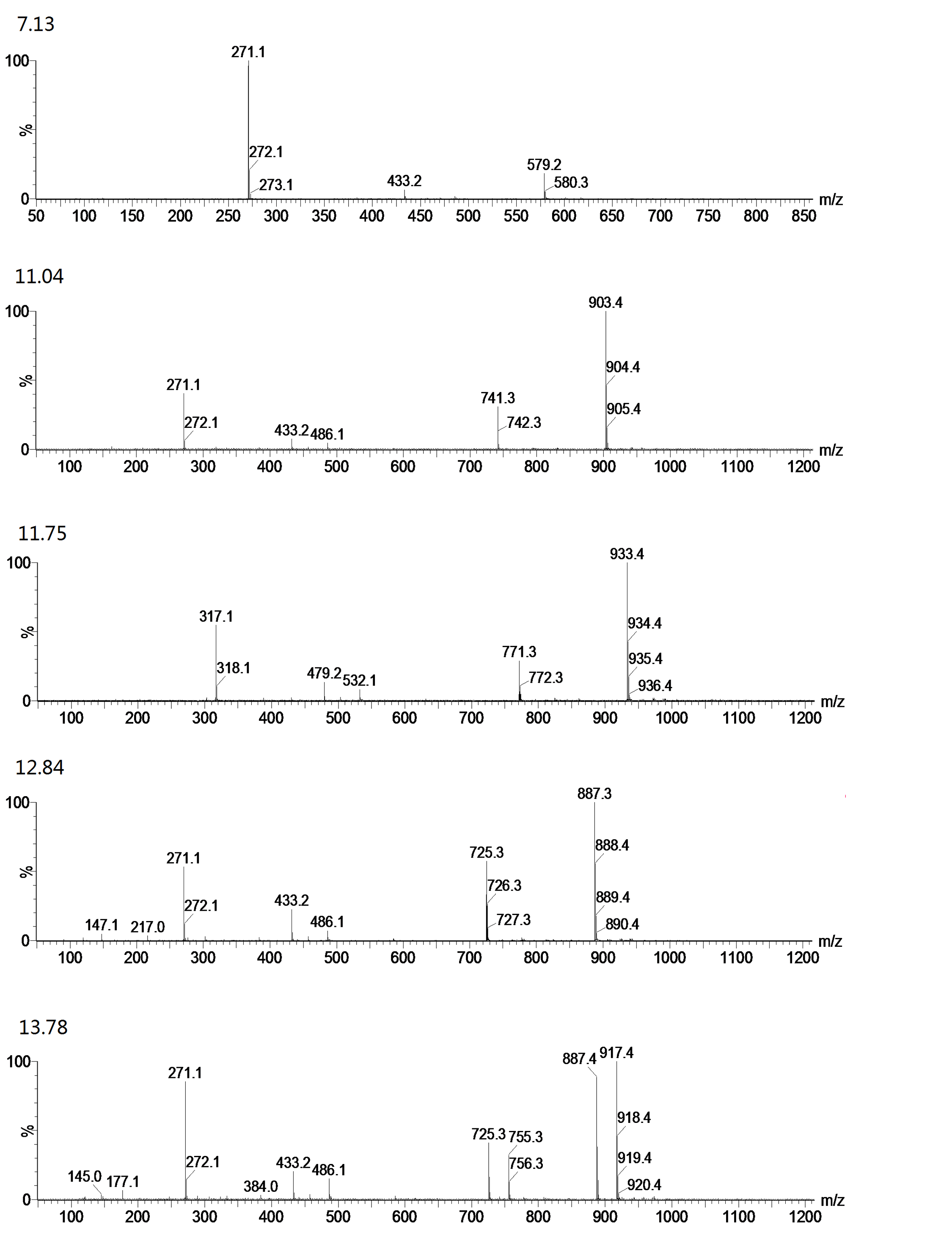

Supplement: S2 Fig — (TIF) [file pone.0191406.s002.tif]

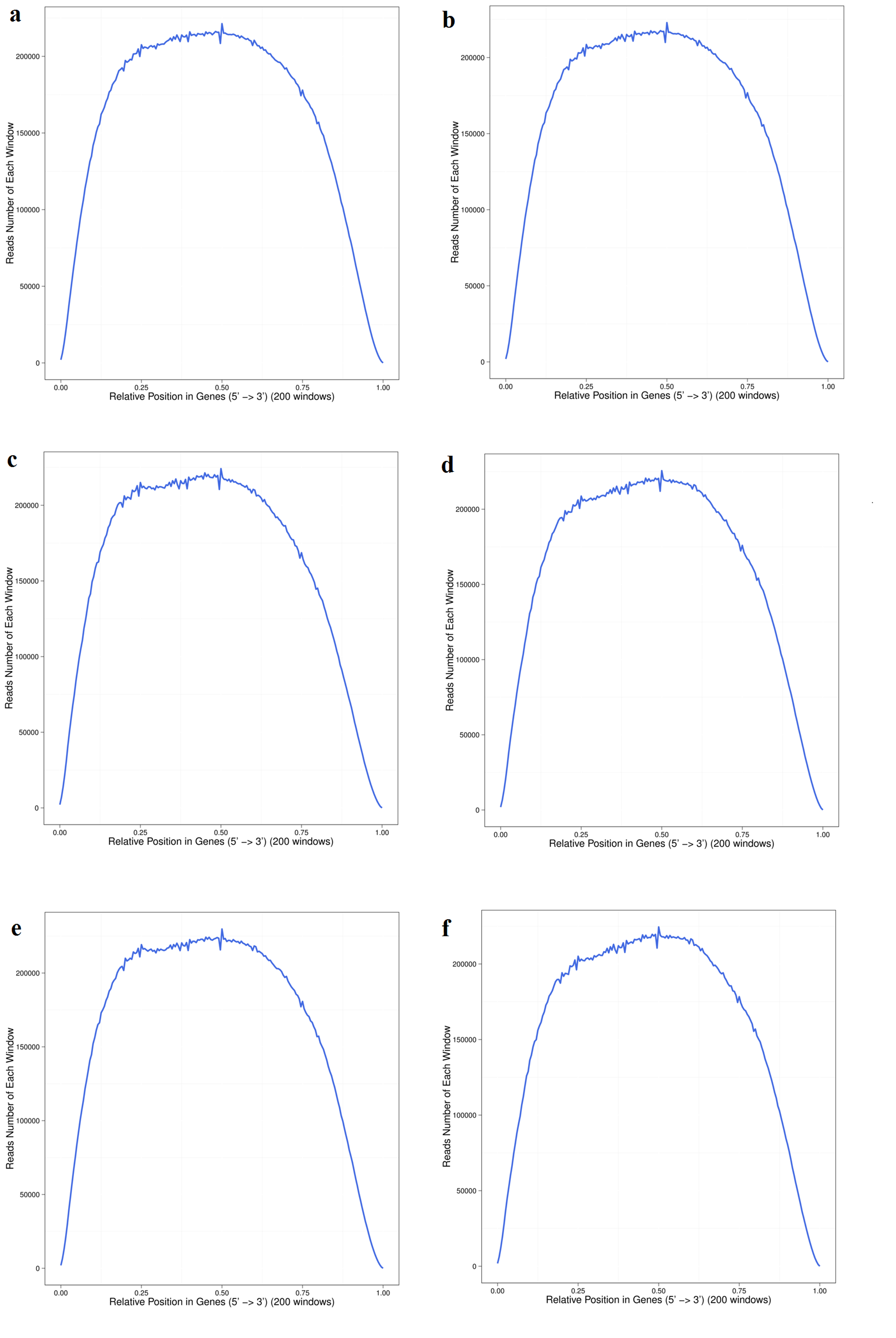

Supplement: S3 Fig — SD140:a,b,c. SD92: d,e,f. (TIF) [file pone.0191406.s003.tif]

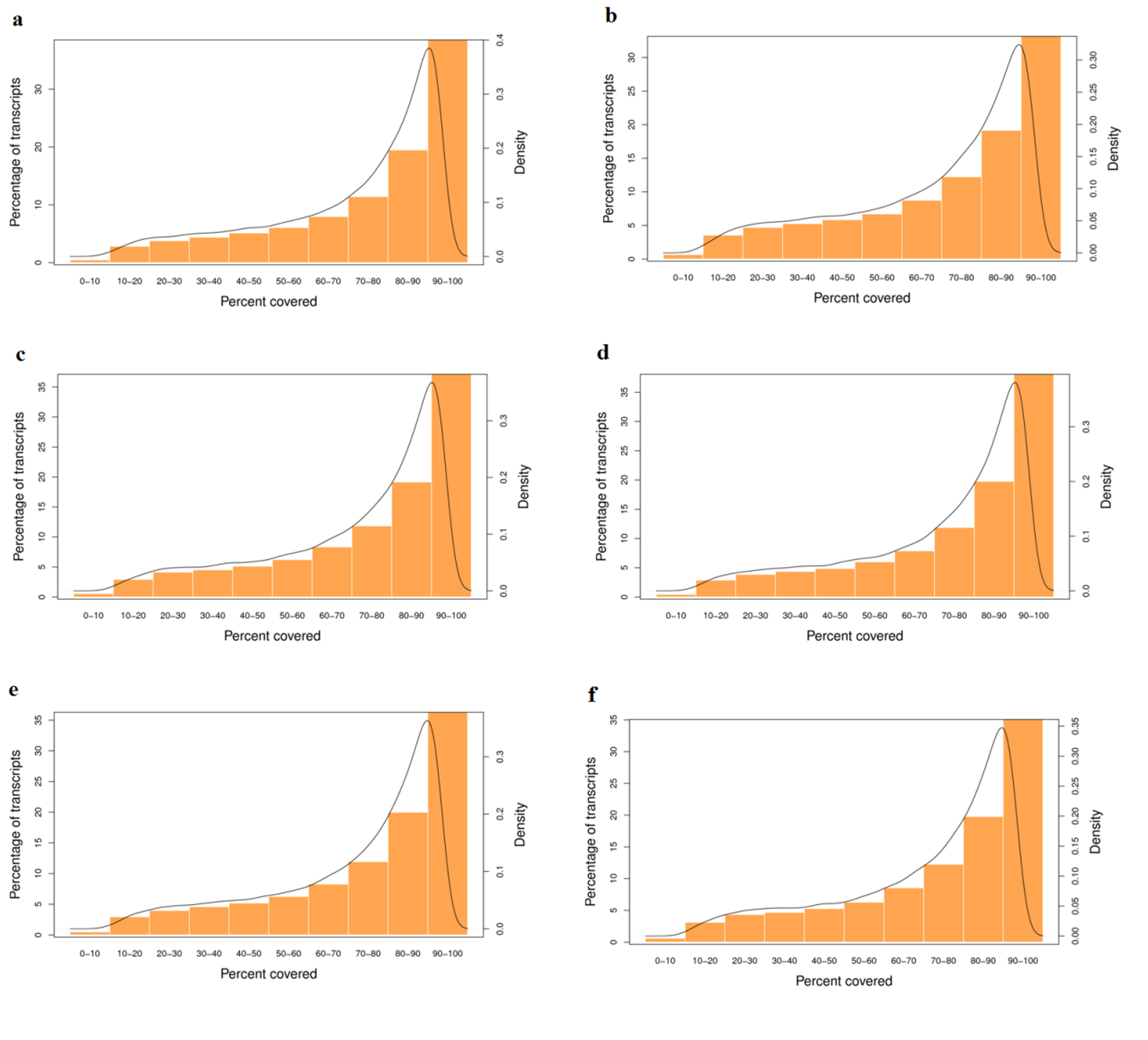

Supplement: S4 Fig — SD140:a,b,c. SD92: d,e,f. (TIF) [file pone.0191406.s004.tif]

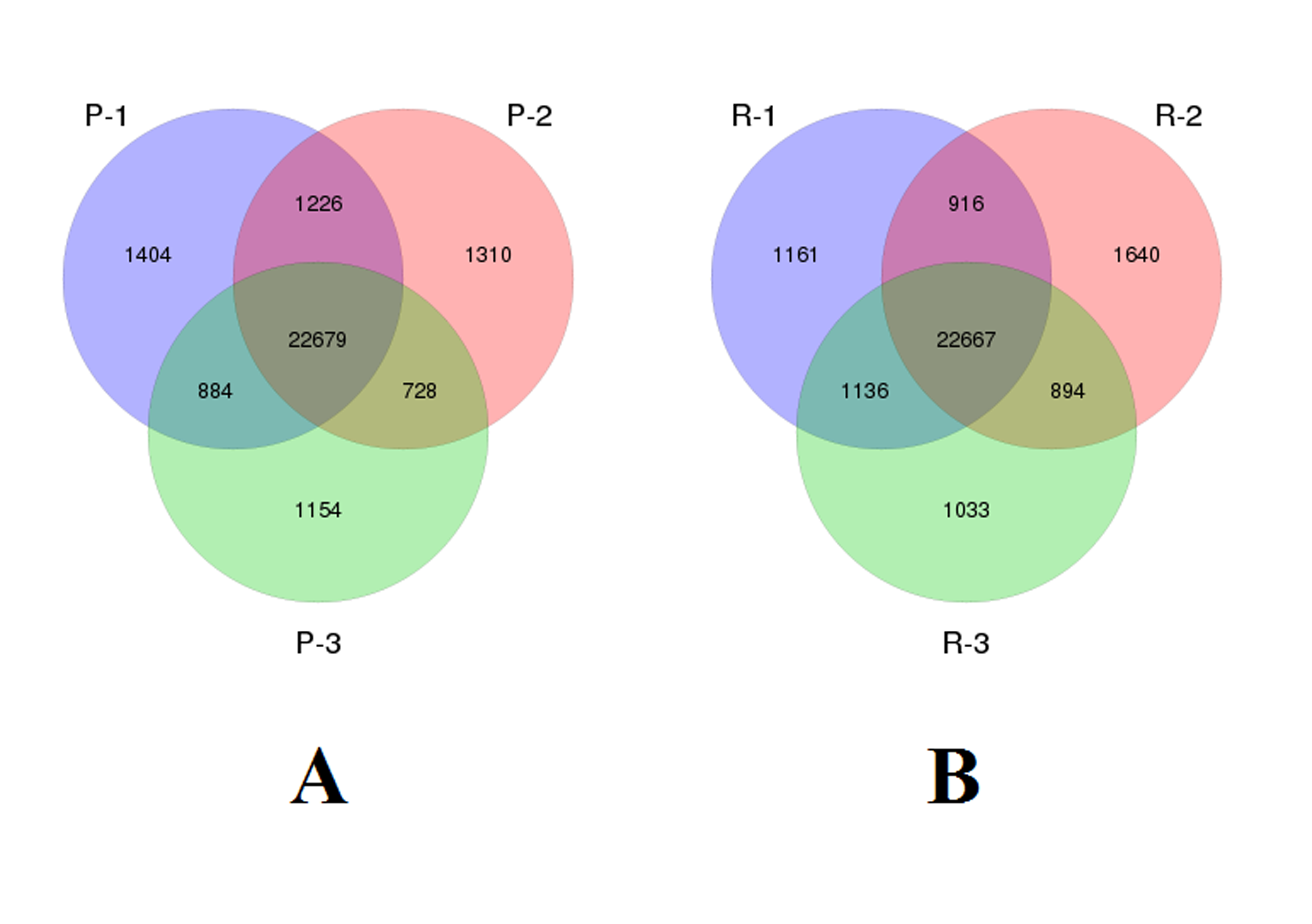

Supplement: S5 Fig — A: SD92, B: SD140. (TIF) [file pone.0191406.s005.tif]

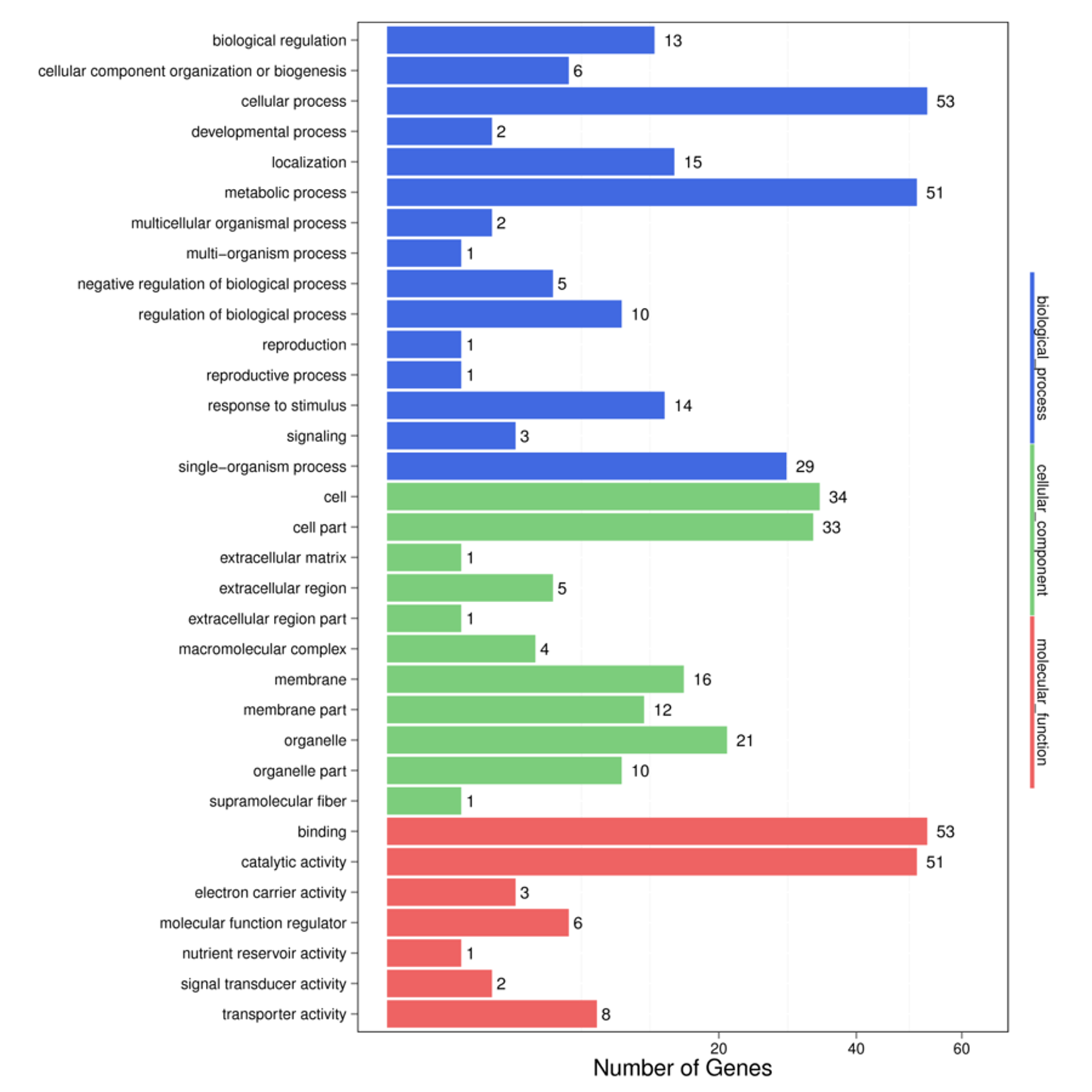

Supplement: S6 Fig — A threshold of corrected p value ≤ 0.001 was used to judge the significantly enriched GO terms in DEGs. (TIF) [file pone.0191406.s006.tif]
