# Supplementary material for: A comparative transcriptome analysis of a wild purple potato and its red mutant provides insight into the mechanism of anthocyanin transformation
Source: PLoS One. 2018 Jan 23;13(1):e0191406. doi: 10.1371/journal.pone.0191406 (PMC5779664; doi:10.1371/journal.pone.0191406)
Supplement: S1 Table — (DOC) [file pone.0191406.s007.doc]

S1 Table List of primers used for validation of the differently expressed genes

| **Gene name** | **Primer** | **Sequence(5′→3′)** |
| --- | --- | --- |
| **PGSC0003DMG400031535** | For | TTAGCGGAGACTTCATTGGA |
|  | Rev | CCACCTGGAGGCTTATGTC |
| **PGSC0003DMG400005969** | For | CCGTTCAATCATCTTCGCT |
|  | Rev | CGGCATCGTAAAGCGTATT |
| **PGSC0003DMG400011950** | For | GCTGGTGCTTGCTCATCTT |
|  | Rev | CCTTCATCCATTTGTCCATCA |
| **PGSC0003DMG400037860** | For | CTTCATCGTATTGGACGCA |
|  | Rev | GCATCCTCTCATCATCCTTT |
| **PGSC0003DMG400021877** | For | GGACAACACACCCATAAGAGTTT |
|  | Rev | GCATTCCAAAGGCTTGAGTAA |
| **PGSC0003DMG400021351** | For | AACGGTCCGAGTTAGCCTATT |
|  | Rev | AATCAAGAATGAGGTGCTCCTT |
| **PGSC0003DMG400007972** | For | CAGGGAATGAACTTGCCAA |
|  | Rev | CAATGGGACTGGGAATGGT |
| **PGSC0003DMG400010713** | For | TCAAGATTTGCCTGTGGTTT |
|  | Rev | TTCTGGATTCAGTTGTTCACCT |
| **PGSC0003DMG400024344** | For | GCCAGAGTCCCTCAACCCA |
|  | Rev | ACAAGTTTTCGCTAGTATAGCAGAGC |
| **PGSC0003DMG400000425** | For | TCAATCCCGAGAGGTTCCT |
|  | Rev | TTCCACCATCACTATTCCCAT |
| **18S** | For | CCTGGTCGGCATCGTTTA |
|  | Rev | CGAACAACTGCGAAAGCAT |
