# Supplementary material for: A comparative transcriptome analysis of a wild purple potato and its red mutant provides insight into the mechanism of anthocyanin transformation
Source: PLoS One. 2018 Jan 23;13(1):e0191406. doi: 10.1371/journal.pone.0191406 (PMC5779664; doi:10.1371/journal.pone.0191406)
Supplement: S2 Table — (DOC) [file pone.0191406.s008.doc]

**S2 Table Summary of mapping results (mapping to genome)**

| **Sample** | **Replication** | **Total raw reads(Mb)** | **Clean reads(Mb)** | **Total mapped ratio** | **Uniquely mapped ratio** |
| --- | --- | --- | --- | --- | --- |
| **SD140** | 1 | 58.78 | 44.95 | 56.75% | 52.73% |
|  | 2 | 55.51 | 44.43 | 61.49% | 57.98% |
|  | 3 | 58.78 | 44.81 | 55.63% | 51.39% |
| **SD92** | 1 | 57.15 | 44.42 | 61.11% | 58.09% |
|  | 2 | 57.14 | 44.15 | 61.37% | 58.31% |
|  | 3 | 58.78 | 44.46 | 56.76% | 52.49% |
