# Supplementary material for: A comparative transcriptome analysis of a wild purple potato and its red mutant provides insight into the mechanism of anthocyanin transformation
Source: PLoS One. 2018 Jan 23;13(1):e0191406. doi: 10.1371/journal.pone.0191406 (PMC5779664; doi:10.1371/journal.pone.0191406)
Supplement: S3 Table — (DOC) [file pone.0191406.s009.doc]

**S3 Table Expression of genes involved in anthocyanin biosynthesis**

| **#Gene** | **Function** | | **SD140** | **SD92** | **Rate of change (%)** | **Pvalue** |
| --- | --- | --- | --- | --- | --- | --- |
| **BGI_novel_G000194** | | phenylalanine ammonia-lyase | 645.08 | 817.80 | -21.12 | 0.40 |
| **PGSC0003DMG400023458** | | phenylalanine ammonia-lyase | 1506.21 | 1395.01 | 7.97 | 0.74 |
| **PGSC0003DMG402021564** | | phenylalanine ammonia-lyase | 1183.71 | 1730.77 | -31.61 | 0.07 |
| **BGI_novel_G001148** | | phenylalanine ammonia-lyase | 61.36 | 68.48 | -10.39 | 0.77 |
| **BGI_novel_G001145** | | phenylalanine ammonia-lyase | 127.31 | 185.62 | -31.41 | 0.27 |
| **BGI_novel_G001146** | | phenylalanine ammonia-lyase | 155.17 | 162.92 | -4.76 | 0.87 |
| **BGI_novel_G001147** | | phenylalanine ammonia-lyase | 238.86 | 322.18 | -25.86 | 0.38 |
| **BGI_novel_G002714** | | phenylalanine ammonia-lyase | 63.75 | 98.51 | -35.29 | 0.09 |
| **PGSC0003DMG400019386** | | phenylalanine ammonia-lyase | 340.16 | 139.33 | 144.13 | 0.01 |
| **PGSC0003DMG400031365** | | phenylalanine ammonia-lyase | 1237.64 | 951.63 | 30.05 | 0.24 |
| **PGSC0003DMG400036104** | | phenylalanine ammonia-lyase | 162.88 | 259.31 | -37.19 | 0.09 |
| **PGSC0003DMG401021549** | | phenylalanine ammonia-lyase | 21212.73 | 30394.67 | -30.21 | 0.11 |
| **PGSC0003DMG402021549** | | phenylalanine ammonia-lyase | 6207.38 | 10809.57 | -42.58 | 0.01 |
| **PGSC0003DMG400031457** | | phenylalanine ammonia-lyase 1 | 331.99 | 447.27 | -25.77 | 0.29 |
| **PGSC0003DMG401030469** | | trans-cinnamate 4-monooxygenase | 1261.67 | 1354.45 | -6.85 | 0.80 |
| **PGSC0003DMG402030469** | | trans-cinnamate 4-monooxygenase | 1354.51 | 1544.61 | -12.31 | 0.55 |
| **PGSC0003DMG401009291** | | 4-coumarate--CoA ligase | 42.16 | 41.20 | 2.34 | 0.93 |
| **PGSC0003DMG400015190** | | 4-coumarate--CoA ligase | 22.91 | 25.85 | -11.39 | 0.71 |
| **PGSC0003DMG400008122** | | 4-coumarate--CoA ligase | 51.90 | 86.25 | -39.83 | 0.01 |
| **PGSC0003DMG400029322** | | 4-coumarate--CoA ligase | 89.99 | 136.82 | -34.23 | 0.06 |
| **BGI_novel_G001112** | | 4-coumarate--CoA ligase | 60.52 | 88.53 | -31.64 | 0.07 |
| **PGSC0003DMG401019891** | | 4-coumarate--CoA ligase 2 | 61.09 | 66.77 | -8.50 | 0.75 |
| **PGSC0003DMG400003155** | | 4-coumarate--CoA ligase 2 | 300.54 | 437.39 | -31.29 | 0.26 |
| **PGSC0003DMG400028929** | | 4-coumarate--CoA ligase 2 | 201.43 | 217.47 | -7.37 | 0.66 |
| **PGSC0003DMG400014223** | | 4-coumarate:coenzyme A ligase | 399.86 | 385.91 | 3.61 | 0.06 |
| **PGSC0003DMG400008551** | | acetyl-CoA carboxylase | 1034.77 | 1277.58 | -19.01 | 0.13 |
| **PGSC0003DMG400028542** | | acetyl-CoA carboxylase | 226.72 | 223.24 | 1.56 | 0.95 |
| **PGSC0003DMG400033054** | | acetyl-CoA carboxylase 1 | 203.94 | 211.08 | -3.38 | 0.89 |
| **PGSC0003DMG400023955** | | acetyl-CoA carboxylase biotin carboxyl carrier protein | 264.62 | 163.55 | 61.80 | 0.09 |
| **PGSC0003DMG401023454** | | acetyl-CoA carboxylase biotin carboxyl carrier protein | 181.36 | 123.23 | 47.17 | 0.09 |
| **PGSC0003DMG400029620** | | chalcone synthase 1B | 654.62 | 546.61 | 19.76 | 0.71 |
| **PGSC0003DMG400019110** | | chalcone synthase 2 | 6002.87 | 6991.90 | -14.15 | 0.60 |
| **PGSC0003DMG400011655** | | chalcone--flavonone isomerase 3 | 1148.02 | 844.17 | 35.99 | 0.40 |
| **BGI_novel_G001772** | | chalcone--flavonone isomerase B | 689.60 | 604.02 | 14.17 | 0.67 |
| **PGSC0003DMG400000425** | | flavonoid 3',5'-hydroxylase | 2136.20 | 3426.67 | -37.66 | 0.02 |
| **PGSC0003DMG400004824** | | flavonoid 3'-monooxygenase | 76.07 | 70.69 | 7.61 | 0.77 |
| **PGSC0003DMG401004779** | | flavonoid 3'-monooxygenase | 60.29 | 61.61 | -2.16 | 0.94 |
| **PGSC0003DMG402004779** | | flavonoid 3'-monooxygenase | 53.25 | 55.76 | -4.49 | 0.87 |
| **PGSC0003DMG400003605** | | dihydroflavonol-4-reductase | 3664.01 | 3958.70 | -7.44 | 0.68 |
| **PGSC0003DMG400015444** | | dihydroflavonol-4-reductase | 96.17 | 84.61 | 13.66 | 0.61 |
| **PGSC0003DMG400022746** | | leucoanthocyanidin dioxygenase | 3420.36 | 3328.75 | 2.75 | 0.93 |
| **PGSC0003DMG401011292** | | leucoanthocyanidin dioxygenase | 19.05 | 25.96 | -26.61 | 0.43 |
| **PGSC0003DMG400011973** | | anthocyanidin 3-O-glucosyltransferase | 89.37 | 100.58 | -11.14 | 0.62 |
| **PGSC0003DMG400024344** | | anthocyanidin 3-O-glucosyltransferase | 3610.01 | 2903.14 | 24.35 | 0.50 |
| **PGSC0003DMG400017737** | | anthocyanidin 3-O-glucosyltransferase 2 | 33.69 | 32.78 | 2.75 | 0.93 |
| **PGSC0003DMG400011971** | | anthocyanidin 3-O-glucosyltransferase 2 | 52.75 | 48.68 | 8.36 | 0.82 |
